# Intestinal Colonization of Multidrug-Resistant Organisms: Identification and Antibiotic Susceptibility Patterns of Bacteria Isolated From Healthy Human Fecal Specimens

**DOI:** 10.7759/cureus.75006

**Published:** 2024-12-02

**Authors:** Vishnu Vandana Waddepally, Sofiya Rabye, Ryhana Bashir, Venkataramana Kandi

**Affiliations:** 1 Microbiology, Vydehi Institute of Medical Sciences and Research Center, Bangalore, IND; 2 Microbiology, Lakshmi Narain Medical College, Bhopal, India; 3 Microbiology, Mansarovar Institute of Medical Sciences, Bhopal, IND; 4 Clinical Microbiology, Prathima Institute of Medical Sciences, Karimnagar, IND

**Keywords:** ampc beta-lactamase, carbapenemase, extended-spectrum beta-lactamase (esbl), methicillin resistance, multidrug-resistant bacteria, multidrug-resistant organisms (mdros), vancomycin resistance

## Abstract

Introduction

Intestinal carriage of multidrug-resistant organisms (MDROs) in healthy populations could amplify resistant bacteria, which may increase the risk of infections by these bacteria in the community and in the hospital. This study investigated the prevalence of colonization of multidrug-resistant (MDR) bacteria in the intestines of healthy individuals in South India.

Methods

A prospective study was conducted for six months at a tertiary care teaching hospital. Two hundred and fifty-five fecal samples collected from healthy individuals were processed according to standard microbiological guidelines. The bacteria (n=291) isolated from the samples were identified and evaluated using phenotypic detection methods for the presence of extended-spectrum beta-lactamase (ESBL), AmpC β-lactamase, carbapenemase, methicillin resistance, and vancomycin resistance.

Results

The prevalence of intestinal carriage of MDR bacteria in healthy populations was 57.04% (166/291). *Klebsiella *(81.92%; 68/83)* *was the most resistant bacterial isolate among the tested organisms. ESBL, AmpC β-lactamase, carbapenemase, and methicillin resistance rates were 34.70% (101/291), 12.37% (36/291), 7.90% (23/291), and 2.06% (6/291), respectively. Both ESBL and AmpC β-lactamase co-producing strains were 3.78% (11/291). Vancomycin resistance was not revealed among the sampled isolates.

Conclusion

The study revealed a high carriage rate of MDROs colonized in the intestines of healthy adults in the community. These results highlight the importance of identifying resistant pathogens through regular surveillance thereby understanding their epidemiology.

## Introduction

The spread of bacteria demonstrating antimicrobial resistance (AMR) has become a global public health crisis, with predictions suggesting 10 million deaths annually by 2050 [1]. Countries have developed national action plans aligned with World Health Organization (WHO) guidelines, emphasizing effective AMR surveillance to track and measure resistance [2]. A key approach is monitoring the colonization of multidrug-resistant organisms (MDROs). This provides a more comprehensive view of AMR strains in community and hospital settings [3]. Asymptomatic carriers may act as reservoirs, increasing transmission risk [4].

Bacteria demonstrating AMR have been frequently identified in hospitalized populations. Interestingly, gram-negative bacteria like *Escherichia coli *(*E*. *coli*)* *and *Klebsiella *species (spp.) colonizing the human intestines are commonly isolated from* *clinical specimens [5,6]. Further, *Staphylococcus aureus *(*S*. *aureus*)* *and *Enterococcus *spp. are frequent gram-positive bacteria isolated from patients' samples.

Indiscriminate use of antimicrobial agents is the primary cause of the development of MDROs. MDROs gain AMR due to the production of certain enzymes like beta-lactamases, extended-spectrum beta-lactamases (ESBLs), carbapenemases, and other mechanisms. Methicillin resistance among *S*. *aureus *strains confers them multidrug resistance [7]. AMR is coded on the genes present on the chromosomes and plasmids. Further, these genes can be transferable within the same and to other bacterial species posing a threat to the emergence of MDROs [5,6].

While significant knowledge exists on MDROs in hospitals, less is known about their prevalence in the community, especially among healthy population. This study aims to assess the colonization rates of resistant bacterial species, among healthy adults in South India, where research on this problem is limited.

## Materials and methods

This prospective study was conducted between July and December 2023 and included urban-dwelling healthy adults between 15 and 55 years old. After obtaining formal informed consent, clinical data and fecal samples were collected from 255 participants. All details regarding demographic information, education, occupation, awareness of antibiotics, antibiotic use, travel history in the past year, and history of animal contact were gathered by providing a questionnaire to the participants. The institution's Ethics committee approved the study, Vydehi Institute of Medical Sciences and Research Center (EC Reg No: VIEC/2023/APP/98 letter dated June 8, 2023).

Inclusion and exclusion criteria

The study included healthy individuals with no history of antibiotic use for at least three months, no hospital admissions in the past year, and no known active infections. All individuals hospitalized within 90 days, who received oral or parenteral antibiotics or chemotherapy in the past 30 days, or who underwent dialysis were excluded from the study.

Sample collection

Fecal samples were collected in a clean wide-mouthed container and transported to the laboratory at 4°C within four hours of collection. All the samples were cultured on blood agar and MacConkey's agar and aerobically incubated at 37°C for 24 hours and bacterial growth was identified using standard bacteriological techniques [8]. Antibiotic susceptibility testing (AST) and phenotypic detection of ESBL, AmpC β-lactamase, and carbapenemase were carried out among Enterobacteriaceae (*E*. *coli*, *Klebsiella *spp., *Enterobacter *spp., and *Citrobacter *spp.) isolates as per the Clinical and Laboratory Standards Institute (CLSI) recommendations [9]. *S*. *aureus *and *Enterococcus *spp. were identified and screened for methicillin and vancomycin resistance.

Antimicrobial susceptibility testing

The Kirby-Bauer disk diffusion method was used to assess the susceptibility patterns of the isolates against different antibiotic groups like aminoglycosides, carbapenems, fluoroquinolones, and cephalosporins. The drugs with standard concentrations tested for Gram-negative bacteria included amoxicillin (30 µg), amoxicillin + clavulanic acid (20/10 µg), aztreonam (30 µg), cefotaxime (30 µg), ceftriaxone (30 µg), ceftazidime (30 µg), cefoxitin (30 µg), cefixime (5 µg), gentamicin (10 µg), amikacin (10 µg), cotrimoxazole (1.25/23.75 µg), ciprofloxacin (5 µg), piperacillin-tazobactam (30/6 µg), imipenem (10 µg), ertapenem (10 µg), and meropenem (10 µg). For Gram-positive bacteria, the tested drugs included penicillin (10 units), oxacillin (1 µg), cotrimoxazole (1.25/23.75 µg), ciprofloxacin (5 µg), tetracycline (30 µg), erythromycin (15 µg), clindamycin (2 µg), gentamicin (10 µg), rifampin (15 µg), vancomycin (30 µg), teicoplanin (30 µg), linezolid (30 µg), daptomycin (30 µg). *E. coli *American Type Culture Collection (ATCC®) 25922, *Klebsiella pneumoniae *ATCC® 700603, *S*.* aureus *ATCC® 29213, and *Enterococcus fecalis *ATCC® 29212 were included as standard control strains. Isolates resistant to at least one antimicrobial agent in three or more antimicrobial categories were considered MDROs [10].

Identification of ESBL-producing strains

ESBL-producing strains were screened based on reduced susceptibility to cefotaxime (30 µg), ceftriaxone (30 µg), and ceftazidime (30 µg) discs with the zone of inhibition ≤27 millimeters (mm), ≤25 mm and ≤22 mm, respectively. Potential ESBL producers were confirmed using the combined disk test (CDT) and the minimum inhibitory concentration (MIC) test with a ceftazidime/cefotaxime+clavulanic acid strip (Hi-media Ezy MIC™ Strip, HiMedia Laboratories Private Limited, India; Triple ESBL detection). *E*. *coli *ATCC® 25922, and *Klebsiella pneumoniae *ATCC® 700603 were used as negative and positive controls, respectively.

Identification of AmpC β-lactamase producing strains

Plasmid-mediated AmpC β-lactamase-producing strains were screened with cefoxitin (30 µg). AmpC β-lactamase production was confirmed in isolates with ≤16 mm zone of inhibition with a cefotetan/cefotetan+cloxacillin MIC test strip. *Klebsiella pneumoniae *ATCC® BAA 1705 and *E*. *coli *ATCC® 25922 were included as positive and negative controls, respectively.

Identification of carbapenemase producers

Gram-negative bacterial isolates that were resistant to one or more carbapenem group antibiotics, and resistant to one or more agents in third-generation cephalosporin were tested for carbapenemase production by a modified carbapenem inactivation method as per CLSI guidelines. *E*. *coli *ATCC® 25922 was used as the control strain.

Identification of methicillin resistance in *S*. *aureus*

*S*.* aureus *isolates resistant to cefoxitin (30 µg) with a zone of inhibition ≤21mm by disc diffusion and MIC >4 µg/mL by VITEK® 2 (bioMérieux, Inc., France) system were identified as methicillin-resistant *S*. *aureus *(MRSA).

Identification of vancomycin resistance in *Enterococcus *spp.

*Enterococcus *species isolates resistant to vancomycin (30 µg) with a zone of inhibition ≤14 mm by disc diffusion and MIC values >8 µg/mL by VITEK® 2 (bioMérieux, Inc.) system were considered vancomycin-resistant enterococci (VRE).

Statistical analysis

All the data were recorded in a Microsoft Office 2019 Excel sheet (Microsoft Corp., Redmond, WA, USA), and statistical analysis was performed using IBM SPSS Statistics for Windows, Version 20 (Released 2011; IBM Corp., Armonk, NY, USA). The results were presented as percentages and proportions, with frequency and percentages used to represent categorical variables.

## Results

Of the 255 healthy adults enrolled in the study, 291 bacterial isolates were identified. Of these, 166 (57.04%) strains were confirmed as MDROs. Analysis of the demographic characters of the people from whom the MDROs were cultured revealed a majority of males (98/166; 59.03%) and individuals ranging from 15 to 55 years. Most study subjects belonged to the 46-55 age group (89/166; 53.61%). Over half (86/166; 51.80%) revealed antibiotic intake in the last six months. Most participants were educated (131/166; 78.91%) and employed (129/166; 77.71%) but lacked awareness of antibiotics (113/166; 68.07%) and failed to complete the course (75/166; 45.18%). Additionally, 22.89% (38/166) gave travel history, and 41.56% (69/166) had contact with pet animals. The demographic and other details of the participants demonstrating MDROs are detailed in Table 1.

**Table 1:** Demographic characteristics of the participants from whom MDROs were isolated

Demographic characteristics	Variable	Total n=166, n (%)
Age (years)	15-30	25 (15.06)
	31-45	52 (31.32)
	46-55	89 (53.61)
Sex	Male	98 (59.03)
	Female	68 (40.96)
Occupation	Employed	129 (77.71)
	Unemployed	37 (22.28)
Education	Illiterate	35 (21.08)
	Literate	131 (78.91)
Prior antibiotic use	4-6 months	86 (51.80)
	>6 months	55 (33.13)
Antibiotic awareness	Yes	53 (31.92)
	No	113 (68.07)
Antibiotic course	Completed the course	91 (54.81)
	Discontinued	75 (45.18)
Self-medication during similar complaints in the future	Consult doctor	101 (60.84)
	Start same medication	65 (39.15)
History of Travel	Yes	38 (22.89)
	No	128 (77.10)
Animal contact	Yes	69 (41.56)
	No	97 (58.43)

Of the 291 bacteria isolated, *E*. *coli *(130/291; 44.67%) was the most frequent gram-negative bacteria, followed by *Klebsiella *spp. (83/291; 28.52%), *Enterobacter *spp. (24/291; 8.24%), and *Citrobacter *spp. (18/291; 6.18%). *S*. *aureus *(26/291; 8.93%) and *Enterococcus *spp. (10/291; 3.43%) were the gram-positive bacterial species isolated. Of these, MDR was noticed more frequently in *Klebsiella *spp. (68/83; 81.92%) followed by *E*. *coli* (76/130; 58.46%), and *Enterobacter *spp. (13/24; 54.16%) as shown in Table 2.

**Table 2:** Characteristics of bacterial colonization and multidrug resistance MDROs: multidrug-resistant organisms; spp.: species

Bacteria	Colonization, n (%)	MDROs, n (%)
*E*. *coli*	130 (44.67)	76 (58.46)
*Klebsiella *spp.	83 (28.52)	68 (81.92)
*Enterobacter *spp.	24 (8.24)	13 (54.16)
*Citrobacter *spp.	18 (6.18)	03 (16.66)
*S*. *aureus*	26 (8.93)	06 (23.07)
*Enterococcus *spp.	10 (3.43)	00 (00)
Total	291 (100)	166 (57.04)

Overall, the prevalence of ESBL (34.70%; 101/291), AmpC β-lactamase (12.37%; 36/291), ESBL and AmpC β-lactamase co-producers (3.78%; 11/291), carbapenemase (7.90%; 23/291) and MRSA (2.06%; 6/291) were observed with no evidence of fecal carriage of VRE. The antibiotic resistance patterns of the isolated bacteria against different antibiotics are detailed in Table 3.

**Table 3:** Resistance profiles of the bacterial isolates against different antibiotics NT: not tested

Antibiotic	*E*. *coli; *Total n=130, n (%)	*Klebsiella; *Total n*=*83, n (%)	*Enterobacter; *Total n=24, n (%)	*Citrobacter; *Total n=18, n (%)	*S*. *aureus; *Total n=26, n (%)	*Enterococcus; *Total n=10, n (%)
Amoxicillin	99 (76)	62 (48)	17 (71)	06 (33)	11 (42)	03 (30)
Amikacin	25 (19)	18 (22)	06 (25)	03 (16)	NT	NT
Amoxiclav	51 (39)	53 (64)	10 (42)	03 (16)	NT	NT
Ciprofloxacin	87 (67)	73 (88)	22 (90)	12 (66)	09 (35)	02 (20)
Ceftriaxone	65 (50)	54 (65)	09 (37)	02 (11)	NT	NT
Cefotaxime	60 (46)	48 (58)	10 (41)	02 (11)	NT	NT
Ceftazidime	58 (44)	48 (57)	09 (37)	02 (11)	NT	NT
Cefepime	38 (29)	32 (39)	04 (16)	NT	NT	NT
Cefoxitin	26 (20)	17 (20)	07 (29)	NT	06 (23)	NT
Gentamicin	56 (43)	54 (65)	15 (62)	04 (22)	03 (12)	03 (30)
Ertapenem	12 (09)	23 (28)	01 (04)	NT	NT	NT
Imipenem	09 (07)	19 (23)	NT	NT	NT	NT
Meropenem	09 (07)	19 (23)	NT	NT	NT	NT
Cotrimoxazole	67 (52)	66 (80)	16 (66)	03 (16)	06 (23)	NT
Tetracycline	92 (71)	61(73)	14 (58)	03 (16)	06 (23)	00 (00)
Piperacillin/Tazobactam	36 (28)	40 (48)	07 (29)	01 (6)	NT	NT
Aztreonam	56 (43)	45 (54)	09 (37)	02 (11)	NT	NT
Erythromycin	NT	NT	NT	NT	10 (38)	01 (10)
Clindamycin	NT	NT	NT	NT	06 (23)	NT
Daptomycin	NT	NT	NT	NT	00 (00)	00 (00)
Linezolid	NT	NT	NT	NT	00 (00)	00 (00)
Penicillin	NT	NT	NT	NT	14 (54)	04 (40)
Rifampicin	NT	NT	NT	NT	00 (00)	00 (00)
Vancomycin	NT	NT	NT	NT	00 (00)	00 (00)

## Discussion

The global prevalence of MDR bacteria among healthy individuals in the community has reached concerning levels, posing a significant public health threat [11]. Although intestinal colonization with MDR Enterobacteriaceae has been documented worldwide, its prevalence in healthy populations varies widely across regions [12,13]. Low- and middle-income countries bear the heaviest burden of antimicrobial-resistant Gram-negative bacteria but lack comprehensive epidemiological data [14]. While numerous studies have focused on the prevalence of MDROs in hospitalized patients in India, there are relatively few investigations examining its prevalence among asymptomatic healthy individuals [15]. Identifying people colonized with MDROs in the community is crucial, as they may serve as reservoirs for transmission. This information is vital for implementing effective infection control and antimicrobial stewardship programs guiding research priorities. Recent studies indicate an increasing trend in MDRO colonization in the community over time [13,16].

The findings of this study showed a high fecal carriage rate (57.04%; 166/291) of MDROs in healthy individuals. This rate was higher than that reported in India, Kuwait, and Ethiopia which documented a carriage of 30%, 30.50%, and 28.80% of MDROs, respectively [16-18]. Other studies conducted among children in rural India (60.60%) and Bangladesh (78%) reported a high prevalence of MDROs in the intestines [19,20]. A relatively high prevalence of MDROs may be attributed to widespread antimicrobial use, including over-the-counter purchases and misuse. Other factors like environmental contamination and the overuse of antibiotics in livestock and agriculture could also contribute to the spread of MDROs and warrant further surveillance.

In this study, Gram-negative bacterial strains exhibited increased resistance to cephalosporins (45%), with considerable resistance observed for cotrimoxazole (54%), ciprofloxacin (72%), and amoxicillin (67%). However, lower resistance was noted against amikacin, piperacillin-tazobactam, and carbapenems. These findings are consistent with results from a study conducted in Nepal [21].

The prevalence of ESBL and AmpC β-lactamase producers in this study was 34.70% (101/291) and 12.37% (36/291), respectively. These findings were similar to previous studies done in India (35.60% and 17.80%), Nepal (25% and 6.40%), and Taiwan (27.40% and 16.10%) for ESBL and AmpC β-lactamase producers, respectively [16,21,22]. Other studies have shown varying rates of ESBL colonization among healthy adults, including 31.40% in Ethiopia, 22% in Southeast Asia, and 38% in Africa [18,23,24]. Contrarily, lower ESBL colonization was reported in asymptomatic individuals in Southern Taiwan (1.90%) and India (9.72%) [25,26]. Besides, much higher rates of ESBL were reported in Tanzania (91.50%), Laos (70%), and Indonesia (76.70%) [27-29]. A multicentric study revealed the average prevalence of intestinal colonization with ESBL-producing Gram-negative bacteria ranging from 6% to 20% in Europe, Eastern Mediterranean, Africa, and 24.5% in the Western Pacific [13]. These differences in colonization rates can be attributed to variations in study populations, geographical location, and other factors like dietary habits, animal contact, antibiotic usage, and international travel.

Eleven (3.78%; 11/291) bacterial strains in the present study were observed to be ESBL and AmpC β-lactamase co-producers. Other studies from Nepal have reported the rates at 42.20% and 1.80% [21,30]. Carbapenemase was detected in 7.90% (23/291) of isolates with *Klebsiella *spp. a major carbapenemase producer in the present study. These findings align with a study from Kuwait, where 7.70% of food industry workers were found to carry carbapenemase-producing bacteria [17]. Low detection rates of 0.33% 0.80% and 2.40% fecal carriage of carbapenemase-producing bacteria have also been observed in studies from Egypt and Ethiopia [18,31,32]. The detection rate of carbapenem resistance of 6.10% in India [18] and 4.26% in China [33] further highlights geographical variation in the prevalence of such bacteria.

The colonization of VRE in healthy adults revealed considerable variation, ranging from 0% to 21% [34]. VRE colonization was not evident in the current study. This was consistent with findings from the United States of America (USA) [35].

Although the nasal cavities are traditionally regarded as the primary site of *S*. *aureus *colonization, studies have shown that the intestine may also be a colonization site. Positive skin cultures by *S*. *aureus *have been associated with intestinal colonization [36]. Furthermore, gastrointestinal MRSA colonization, even in the absence of nasal carriage was observed in one of every three intestinal carriers. This has been reported as a potential source for MRSA transmission and provides a rationale for performing intestinal screening for surveillance. The detection rate of intestinal carriage for *S*. *aureus *and MRSA in healthy individuals was 20% and 9%, respectively [37]. In our study 2.06% (6/291) gastrointestinal MRSA colonization was noted, whereas 3.80% and 7.80% were reported from Taiwan and Hong Kong, respectively [22,38].

Several risk factors attributed to the acquisition of resistant flora include regular contact with livestock, consumption of contaminated food, international travel, previous hospitalization, and misuse of antibiotics. In our study, participants who had taken antibiotics during the past six months were colonized with MDROs. Other risk factors included lower levels of antibiotic knowledge and tended to repeat the same medication during illness. There has been uncertainty over the effect of age and employment on antibiotic resistance [17].

Study limitations

The study mainly used conventional culture-based isolation, identification, and phenotypic techniques to detect MDROs. The results obtained from this study may have lower sensitivity compared to molecular methods. Additionally, recall bias, wherein the study participants fail to accurately remember a past event or experience, would have affected the accuracy of self-reported data on prior antibiotic usage. The study did not explore the source and factors influencing the colonization of MDROs.

## Conclusions

Given the extent of the public health scare due to microbial drug resistance, the present study spotlights a significantly high prevalence of MDROs in the community. Healthy and asymptomatic colonization of MDROs potentially plays a significant role in spreading AMR, which could influence the hospital epidemiology of such bacterial species. Increasing gut colonization of MDROs warrants clinicians to consider the risk of endogenous infections caused by these difficult-to-treat pathogens. Ongoing surveillance and more stringent regulation of antibiotic use are essential to curb the spread of AMR. Public awareness campaigns on the responsible use of antibiotics are crucial in preventing further escalation of resistance in human microbiota.

## References

[1] Medina M-J, Legido-Quigley H, Hsu LY (2020). Antimicrobial resistance in one health. Global health security. Recognizing vulnerabilities, creating opportunities.

[2] (2024). WHO: Antimicrobial resistance. https://www.who.int/news-room/fact-sheets/detail/antimicrobial-resistance?gad_source=1&gclid=Cj0KCQiAuou6BhDhARIsAIfgrn5RMl85pqyh69UQJXEifNQkqub9tFc8aIE5gSmGr_y3EsBBB9wM2sgaAjWbEALw_wcB.

[3] Worldwide antimicrobial resistance national/international network group (WARNING) collaborators (2023). Ten golden rules for optimal antibiotic use in hospital settings: the WARNING call to action. World J Emerg Surg.

[4] Woerther PL, Burdet C, Chachaty E, Andremont A (2013). Trends in human fecal carriage of extended-spectrum β-lactamases in the community: toward the globalization of CTX-M. Clin Microbiol Rev.

[5] Kandi V, Shahapur PR, Suvvari TK (2024). Molecular characterization of Escherichia coli causing urinary tract infections through next-generation sequencing: a comprehensive analysis of serotypes, sequence types, and antimicrobial and virulence genes. Cureus.

[6] Moses VK, Kandi V, Bharadwaj VG, Suvvari TK, Podaralla E (2024). Molecular characterization of Klebsiella pneumoniae clinical isolates through whole-genome sequencing: a comprehensive analysis of serotypes, sequence types, and antimicrobial and virulence genes. Cureus.

[7] Handa VL, Patel BN, Bhattacharya DA, Kothari RK, Kavathia DG, Vyas BR (2024). A study of antibiotic resistance pattern of clinical bacterial pathogens isolated from patients in a tertiary care hospital. Front Microbiol.

[8] Forbes BA, Sahm DF, Weissfeld AS (2007). Bailey & Scott's Diagnostic Microbiology. https://books.google.co.in/books/about/Bailey_Scott_s_Diagnostic_Microbiology.html?id=URA8ngEACAAJ&redir_esc=y.

[9] (2024). Clinical and Laboratory Standards Institute: performance standards for antimicrobial susceptibility testing; thirtieth informational supplement. M100-S30. https://www.nih.org.pk/wp-content/uploads/2021/02/CLSI-2020.pdf.

[10] Magiorakos AP, Srinivasan A, Carey RB (2012). Multidrug-resistant, extensively drug-resistant and pandrug-resistant bacteria: an international expert proposal for interim standard definitions for acquired resistance. Clin Microbiol Infect.

[11] van Duin D, Paterson DL (2020). Multidrug-resistant bacteria in the community: an update. Infect Dis Clin North Am.

[12] Campos-Madueno EI, Moradi M, Eddoubaji Y (2023). Intestinal colonization with multidrug-resistant Enterobacterales: screening, epidemiology, clinical impact, and strategies to decolonize carriers. Eur J Clin Microbiol Infect Dis.

[13] Bezabih YM, Sabiiti W, Alamneh E, Bezabih A, Peterson GM, Bezabhe WM, Roujeinikova A (2021). The global prevalence and trend of human intestinal carriage of ESBL-producing Escherichia coli in the community. J Antimicrob Chemother.

[14] Antimicrobial Resistance Collaborators (2022). Global burden of bacterial antimicrobial resistance in 2019: a systematic analysis. Lancet.

[15] Arum N, Ghafur A, Kazi M (2022). Prevalence of faecal carriage of carbapenemase producing Enterobacteriaceae in healthy Indian subjects from the community. Indian J Med Microbiol.

[16] Antony S, Ravichandran K, Kanungo R (2018). Multidrug-resistant Enterobacteriaceae colonising the gut of adult rural population in South India. Indian J Med Microbiol.

[17] Moghnia OH, Rotimi VO, Al-Sweih NA (2021). Monitoring antibiotic resistance profiles of faecal isolates of Enterobacteriaceae and the prevalence of carbapenem-resistant isolates among food handlers in Kuwait. J Glob Antimicrob Resist.

[18] Abera D, Negash AA, Fentaw S (2024). High prevalence of colonization with extended-spectrum β-lactamase-producing and multidrug-resistant Enterobacterales in the community in Addis Ababa Ethiopia: risk factors, carbapenem resistance, and molecular characterization. BMC Microbiol.

[19] Sandhu R, Aggarwal A, Sayal P, Kumar S (2019). Intestinal carriage of drug‑resistant Gram‑negative bacteria belonging to family Enterobacteriaceae in children aged 3-14 years: an emerging threat. Int J Health Allied Sci.

[20] Monira S, Shabnam SA, Ali SI (2017). Multi-drug resistant pathogenic bacteria in the gut of young children in Bangladesh. Gut Pathog.

[21] Mandal DK, Sah SK, Mishra SK (2020). Carriage of extended-spectrum-β-lactamase- and AmpC-β-lactamase-producing Enterobacteriaceae (ESBL-PE) in healthy community and outpatient department (OPD) patients in Nepal. Can J Infect Dis Med Microbiol.

[22] Huang YS, Lai LC, Chen YA (2020). Colonization with multidrug-resistant organisms among healthy adults in the community setting: prevalence, risk factors, and composition of gut microbiome. Front Microbiol.

[23] Karanika S, Karantanos T, Arvanitis M, Grigoras C, Mylonakis E (2016). Fecal colonization with extended-spectrum beta-lactamase-producing Enterobacteriaceae and risk factors among healthy individuals: a systematic review and metaanalysis. Clin Infect Dis.

[24] Ouchar Mahamat O, Tidjani A, Lounnas M (2019). Fecal carriage of extended-spectrum β-lactamase-producing Enterobacteriaceae in hospital and community settings in Chad. Antimicrob Resist Infect Control.

[25] Wu PC, Wang JL, Hsueh PR (2019). Prevalence and risk factors for colonization by extended-spectrum β-lactamase-producing or ST 131 Escherichia coli among asymptomatic adults in community settings in Southern Taiwan. Infect Drug Resist.

[26] Aruna S (2018). Incidence of extended spectrum beta lactamase producing Escherichia coli and Klebsiella pneumoniae from faecal samples in patients and healthy subjects. Indian J Microbiol Res.

[27] Büdel T, Kuenzli E, Clément M (2019). Polyclonal gut colonization with extended-spectrum cephalosporin- and/or colistin-resistant Enterobacteriaceae: a normal status for hotel employees on the island of Zanzibar, Tanzania. J Antimicrob Chemother.

[28] Moser AI, Kuenzli E, Campos-Madueno EI (2021). Antimicrobial-resistant Escherichia coli strains and their plasmids in people, poultry, and chicken meat in Laos. Front Microbiol.

[29] Hamid F, Munawir M, Amaruddin AI (2020). Prevalence of fecal carriage of extended-spectrum-lactamase-producing Enterobacteriaceae among school children in South Sulawesi, Indonesia. Arch Clin Infect Dis.

[30] Hosuru Subramanya S, Bairy I, Nayak N, Padukone S, Sathian B, Gokhale S (2019). Low rate of gut colonization by extended-spectrum β-lactamase producing Enterobacteriaceae in HIV infected persons as compared to healthy individuals in Nepal. PLoS One.

[31] Sayed AM, Behiry IK, Elsherief RH, Elsir SA (2017). Detection of carbapenemase-producing Enterobacteriaceae in rectal surveillance cultures in non-hospitalized patients. J Anal Sci Technol.

[32] Amare A, Eshetie S, Kasew D, Moges F (2022). High prevalence of fecal carriage of Extended-spectrum beta-lactamase and carbapenemase-producing Enterobacteriaceae among food handlers at the University of Gondar, Northwest Ethiopia. PLoS One.

[33] Li Y, Ma L, Ding X, Zhang R (2023). Fecal carriage and genetic characteristics of carbapenem-resistant enterobacterales among adults from four provinces of China. Front Epidemiol.

[34] Hannaoui I, Barguigua A, Serray B, El Mdaghri N, Timinouni M, Ait Chaoui A, El Azhari M (2016). Intestinal carriage of vancomycin-resistant enterococci in a community setting in Casablanca, Morocco. J Glob Antimicrob Resist.

[35] Decker BK, Lau AF, Dekker JP (2018). Healthcare personnel intestinal colonization with multidrug-resistant organisms. Clin Microbiol Infect.

[36] Piewngam P, Otto M (2024). Staphylococcus aureus colonisation and strategies for decolonisation. Lancet Microbe.

[37] Acton DS, Plat-Sinnige MJ, van Wamel W, de Groot N, van Belkum A (2009). Intestinal carriage of Staphylococcus aureus: how does its frequency compare with that of nasal carriage and what is its clinical impact?. Eur J Clin Microbiol Infect Dis.

[38] Wong SC, Chen JH, So SY, Ho PL, Yuen KY, Cheng VC (2022). Gastrointestinal colonization of meticillin-resistant Staphylococcus aureus: an unrecognized burden upon hospital infection control. J Hosp Infect.

